# Histologic Definition of Enhancing Core and FLAIR Hyperintensity Region of Glioblastoma, IDH-Wild Type: A Clinico-Pathologic Study on a Single-Institution Series

**DOI:** 10.3390/brainsci13020248

**Published:** 2023-01-31

**Authors:** Giuseppe Broggi, Roberto Altieri, Valeria Barresi, Francesco Certo, Giuseppe Maria Vincenzo Barbagallo, Magda Zanelli, Andrea Palicelli, Gaetano Magro, Rosario Caltabiano

**Affiliations:** 1 Department of Medical and Surgical Sciences and Advanced Technologies “G. F. Ingrassia”, Anatomic Pathology, University of Catania, 95123 Catania, Italy; 2 Department of Neurological Surgery, Policlinico “G. Rodolico-S. Marco” University Hospital, 95123 Catania, Italy; 3 Interdisciplinary Research Center on Brain Tumors Diagnosis and Treatment, University of Catania, 95123 Catania, Italy; 4 Department of Neuroscience “Rita Levi Montalcini”, University of Turin, 10124 Turin, Italy; 5 Department of Diagnostics and Public Health, Section of Anatomic Pathology, University of Verona, 37134 Verona, Italy; 6 Pathology Unit, Azienda USL-IRCCS di Reggio Emilia, 42123 Reggio Emilia, Italy

**Keywords:** glioblastoma, enhancing core, FLAIR, histopathology

## Abstract

The extent of resection beyond the enhancing core (EC) in glioblastoma IDH-wild type (GBM, IDHwt) is one of the most debated topics in neuro-oncology. Indeed, it has been demonstrated that local disease recurrence often arises in peritumoral areas and that radiologically-defined FLAIR hyperintensity areas of GBM IDHwt are often visible beyond the conventional EC. Therefore, the need to extend the surgical resection also to the FLAIR hyperintensity areas is a matter of debate. Since little is known about the histological composition of FLAIR hyperintensity regions, in this study we aimed to provide a comprehensive description of the histological features of EC and FLAIR hyperintensity regions sampled intraoperatively using neuronavigation and 5-aminolevulinic acid (5-ALA) fluorescence, in 33 patients with GBM, IDHwt. Assessing a total 109 histological samples, we found that FLAIR areas consisted in: (i) fragments of white matter focally to diffusely infiltrated by tumor cells in 76% of cases; (ii) a mixture of white matter with reactive astrogliosis and grey matter with perineuronal satellitosis in 15% and (iii) tumor tissue in 9%. A deeper knowledge of the histology of FLAIR hyperintensity areas in GBM, IDH-wt may serve to better guide neurosurgeons on the choice of the most appropriate surgical approach in patients with this neoplasm.

## 1. Introduction

Glioblastoma IDH-wild type (GBM, IDHwt) is the most frequent malignant primary tumor of the central nervous system (CNS) in adults [1,2]. In spite of the advances in neurosurgery, neuropathology and neuro-oncology, it exhibits a uniformly poor prognosis characterized by low survival rates and poor response to the currently available therapies including surgery and chemo/radiotherapy [1,2,3]. In the past, the main goal of surgical resection of GBM, IDHwt was the complete resection of the enhancing core (EC) of the tumor; however, the evidence that local recurrences often originate from the peritumoral zone suggests that “what was going on around the tumor” should also be considered [4,5,6,7,8,9]. On magnetic resonance imaging (MRI), T2-weighted (T2w) sequences identify the different water contents of the cerebral parenchyma; in particular, fluid-attenuated inversion recovery (FLAIR) T2wimages better point out pathological processes, suppressing the T2 signal from cerebrospinal fluid (CSF) [10,11]. Since FLAIR hyperintensity areas are often visible beyond the conventional EC of GBM, a debated issue is whether the surgical resection should also be extended to the FLAIR hyperintensity area, performing a so-called “FLAIRectomy”, and whether this surgical procedure could be of any benefit for the patient’s outcome [4,5,6,7,8,9]. The morphology and the molecular landscape of GBM, IDHwt have been extensively investigated [12,13]; however, little is known about the histological composition of the peritumoral area that corresponds to what is radiologically defined as the FLAIR hyperintensity zone. To the best of our knowledge, relatively few studies have correlated the MRI and histological features of the FLAIR hyperintensity zone [14,15,16]. Therefore, the aim of the present study is to histologically characterize the FLAIR hyperintensity zone, describing the different histological features of both ECs and FLAIR hyperintensity areas in a single-institution series of 33 patients. A deeper knowledge of the tissue composition of these areas could provide useful information to modify therapeutic strategies in these patients.

## 2. Materials and Methods

The present research was in accordance with the Declaration of Helsinki and obtained the approval of the local ethics committee, Catania 1 (CE 165/2015/PO). All the patients involved gave their written informed consent. Inclusion criteria were: (i) histologically proven GBM, IDH-wt; (ii) age > 18 years; (iii) intraoperative sampling of EC and FLAIR zone detected using neuronavigation and 5-aminolevulinic acid (5-ALA) fluorescence.

We prospectively collected tissue samples in different brain tumor regions at the Neurosurgical Unit of the University of Catania between January 2020 and December 2021. We then retrospectively retrieved all cases with a histopathologically- and molecularly-proven diagnosis of “de novo” WHO grade 4 GBM, IDHwt.

The pre-operative and surgical protocols used to selectively identify and sample the different tumor areas foresaw an intraoperative multimodal imaging approach that was previously described [4,17,18]. Particularly, the intraoperative fluorescence with 5-ALA was evaluated using a surgical microscope with 400 nm filter (Carl Zeiss; Kinevo 900, Oberkochen, Germany) by three trained neurosurgeons (G.M.V.B., F.C. and R.A.) during the resection or after the sampling in cases of frameless neuronavigated biopsy. Screenshots of neuronavigation were registered and collected in order to identify the sites of every sample.

Tissue specimens were formalin-fixed, paraffin-embedded, cut to 2–3 microns and stained with hematoxylin and eosin (H&E). A total of 109 histological samples (52 taken from ECs and 57 from FLAIR hyperintensity regions) were evaluated by three pathologists with expertise in neuropathology (G.B., G.M. and R.C.). The Fisher exact test was performed to evaluate the differences in the distribution of histological features between EC and FLAIR hyperintensity regions. A *p*-value of < 0.05 was considered as statistically significant.

## 3. Results

**Figure 1:**
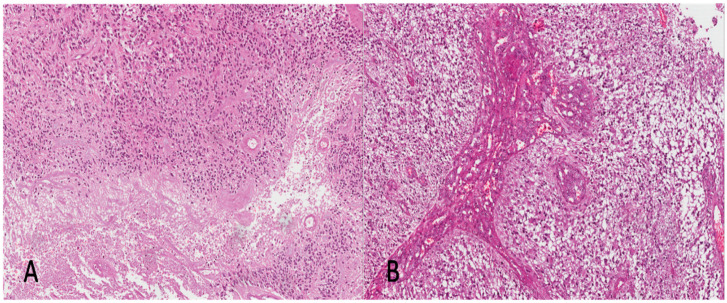
Histopathology of EC. All surgical samples from the ECs exhibited the conventional morphological features of GBM, IDHwt, consisting of hypercellular astrocytic neoplasms with necrosis (**A**) and/or MVP (**B**) ((**A**,**B**) H&E; original magnifications 100×).

Of the 58 patients surgically treated for CNS neoplasms in that period, 33 (20 males and 13 females with a mean age at diagnosis of 56 years) met the inclusion criteria and were included in the study. Tumors were located at the temporal lobe (*n* = 13), frontal lobe (*n* = 9), parietal lobe (*n* = 8), occipital lobe (*n* = 2) and corpus callosum (*n* = 1). Furthermore, 22 (66.6%) underwent resection, while 11 (33.3%) underwent biopsy. All the histologically examined samples exhibited 5-ALA fluorescence; the specimens from the EC showed a lava-like fluorescence, whereas those from the FLAIR hyperintensity zone had a faint fluorescence. In 29/33 (88%) cases, the samples from ECs exhibited classic GBM morphology, consisting of hypercellularity, increased mitotic activity, necrosis (Figure 1A) and/or microvascular proliferation (MVP) (Figure 1B). In 4/33 cases (12%), the specimens from the ECs showed hypercellular and mitotically-active high-grade diffuse astrocytic tumors, IDHwt, lacking both necrosis and MVP. In these cases, the diagnosis of GBM was based on molecular criteria: 3/4 tumors exhibited *EGFR* amplification, combined loss of chromosome 10 and gain of chromosome 7 (7+/10− phenotype) and *TERT* promoter mutation, while *EGFR* amplification and 7+/10− phenotype was found in the remaining case.

**Figure 2:**
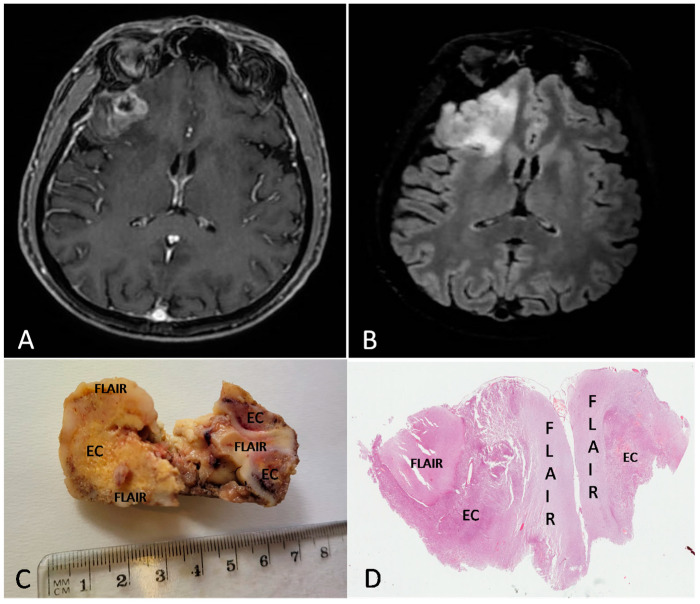
Preoperative MRI from a 57-year-old man. (**A**) Axial section of a T1w sequence with gadolinium showing a left frontal tumor with necrotic core and ring enhancement. (**B**) FLAIR sequence showing a hyperintensity beyond the EC. (**C**,**D**) Gross image (**C**) and histologic low magnification (**D**) showing both EC and FLAIR region from the excised mass ((**D**) H&E; original magnification 25×).

**Figure 3:**
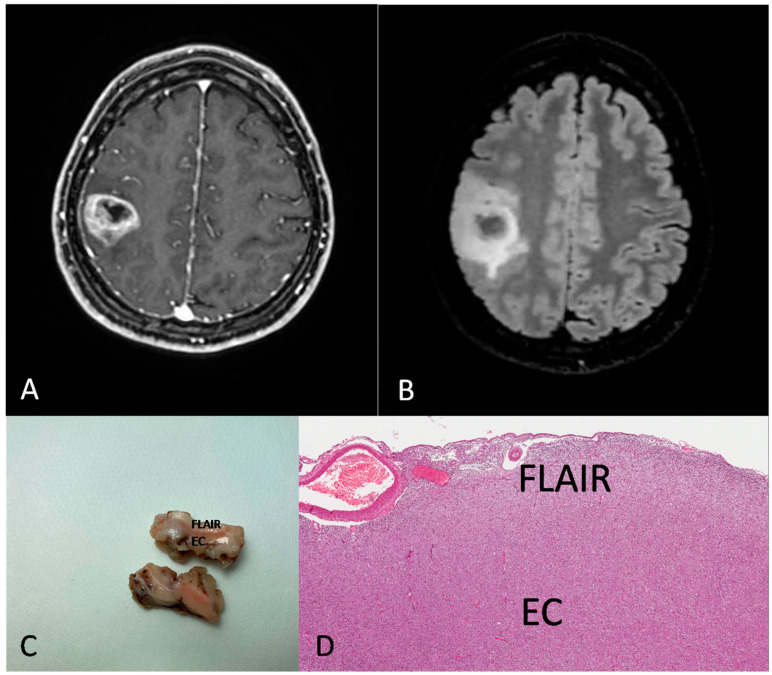
Preoperative MRI from a 31-year-old woman. (**A**) Axial section of a T1-weighted sequence with gadolinium showing a rolandic tumor with necrotic core and ring enhancement. (**B**) FLAIR sequence showing a hyperintensity beyond the EC. (**C**,**D**) Gross image (**C**) and histological medium magnification (**D**) showing both EC and FLAIR region from the excised mass ((**D**) H&E; original magnification 50×).

Figure 2 and Figure 3 show the MRI features, the gross findings and the histopathology of two cases from our series.

**Figure 4:**
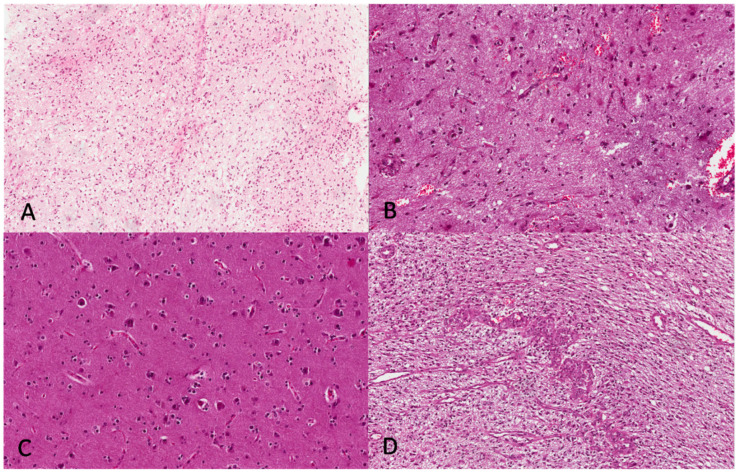
Histopathology of FLAIR hyperintensity region. (**A**) Focal to diffuse infiltration of white matter by tumor cells is seen (H&E; original magnification 100×). (**B**) Reactive astrogliosis of the white matter with gemistocytic features is shown (H&E; original magnification 200×). (**C**) Grey matter exhibiting perineuronal satellitosis (H&E; original magnification 200×). (**D**) A minority of samples show GBM morphology with foci of MVP (H&E; original magnification 100×).

The histological examination of the specimens from FLAIR hyperintensity areas showed: (i) fragments of white matter focally to diffusely infiltrated by tumor cells, in the absence of necrosis and MVP, in 25/33 cases (76%) (Figure 4A); (ii) a mixture of white matter with reactive astrogliosis (Figure 4B) and grey matter exhibiting perineuronal satellitosis (Figure 4C), in 5/33 cases (15%) and (iii) viable tumor tissue with necrosis and MVP (Figure 4D) in 3/33 cases (9%). The Fisher exact test exhibited statistically significant differences in the histological composition between EC and FLAIR regions (*p* = 0.0000) (Table 1). 

## 4. Discussion

Nowadays, a highly debated issue in neuro-oncology is whether surgical resection of GBM, IDHwt should be extended beyond the EC [4,5,6,7,8,9]. Our research group was the first to use the term “FLAIRectomy” to define the resection of the FLAIR hyperintensity area around the EC [4]. Li et al., instead, first focused on the peritumoral infiltrated FLAIR hyperintensity areas, showing that an extent of resection (EOR) >53% of this region was associated with a better prognosis [8], and their findings were confirmed by Pessina et al. [19]. By subdividing the patients into two groups based on the Volume _FLAIR_/Volume _EC_ ratio (Volume _FLAIR_/Volume _EC_ < 10 are defined as the “proliferation-dominant” subtype, Volume _FLAIR_/Volume _EC_ > 10 are defined as “diffusion-dominant” subtypes), some researchers demonstrated that surgical resection of the FLAIR zone leads to survival improvement in patients with “proliferation-dominant” grade 4 astrocytomas, IDH-mutant [20]. However, a multicentric study demonstrated that supramarginal resection improved survival in patients with GBM, IDHwt of the “diffusion dominant” type [9,21]. Recently, Haddad et al. also stated that maximal safe resection of both EC and FLAIR regions provided a survival benefit to patients with GBM [7].

It is widely demonstrated that 5-ALA fluorescence is a useful tool to obtain a supramaximal resection; the intraoperative fluorescence generally overcomes the boundaries of the T1 nodule and it is also known that the range of fluorescence differs between EN and FLAIR areas but there is no clear evidence about the precise spatial correlation between neuroradiological and intraoperative data [18,22,23,24].

Clarifying the pathological features and the biological role of FLAIR hyperintensity zone might be useful to explain the observed survival improvement in GBM patients treated with “FLAIRectomy” [5,25]. Indeed, although it was supposed that FLAIR hyperintensity areas in GBM contain infiltrative tumor cells, there are relatively few literature evidence that systematically define these peritumoral regions from a histopathological point of view [13,14,15]. On an autoptic series, Yamahara et al. showed a significant amount of tumor cells several millimeters beyond the EC in MRI [14]; however, this study had several limitations, including the low number of brains examined (only seven), the comparison of a death brain with an imaging performed much earlier and the lack of consideration of FLAIR sequences. Barajas and colleagues overcame the limitation of the previous study, by examining 119 tissue samples taken during stereotactic biopsies [15]. They showed that tumor cells were present in more than 80% of non-enhancing tumor tissue, challenging the widely held concept that FLAIR hyperintense regions had limited malignant potential [15]. However, this paper was especially focused on the neuroradiological findings and there was no specific characterization of the histological features. Gill et al. evaluated the specimens taken from EC and FLAIR regions of 69 patients affected by GBM [16]. The two different areas were identified with the aid of neuronavigation alone. Pathological analysis showed that the cellular density was significantly different between the two zones (EC samples > FLAIR) [16]. In addition, ECs contained glomeruloid-type vascular proliferation and necrosis, whereas the FLAIR regions exhibited an overall morphology consistent with that of diffusely infiltrating gliomas [16]. We believe that the methods used in this study could determine a sampling bias.

The present study first provides readers with a histological “definition” of FLAIR hyperintensity areas, showing that these regions, which until now have represented a purely radiological concept, exhibit tumor cell infiltration or classical GBM features in 85% of cases. In addition, our research group recently demonstrated a similar amount of cancer stem cells in EC and FLAIR hyperintensity regions of GBM, IDHwt [18], emphasizing that GBM peritumoral zones actively contribute to the aggressive biological behavior of this neoplasm. Our results are partially in line with those from Barajas et al. who demonstrated that neoplastic cells could be found in more than 80% of non-enhancing GBM tissues from their cohort [15] but did not report further histological qualitative differences; we overcame these limitations demonstrating that, according to our data, FLAIR hyperintensity regions rarely (less than 10% of cases) exhibited viable tumor tissue with “conventional” GBM morphology (necrosis and/or MVP), while white matter tissue with at least focal tumor infiltration and absence of necrosis and MVP were seen in the majority (76%) of cases. In addition, the histology of FLAIR areas in which no tumor infiltration was found, consisted of reactive astrogliosis with gemistocytes and perineuronal satellitosis. In this regard, we believe that the histologic/biological concept of perineuronal satellitosis deserves a brief discussion. This histological feature, first described on unaffected peripheral nervous tissue by Santiago Ramón y Cajal in 1899 as “satellitosis” [26] and later renamed “perineuronal satellitosis” in 1930 [27], consists of small aggregates of glial cells surrounding the cell body and the dendrites of neurons [28]. Since its first description, perineuronal satellitosis has been widely reported both in aged and young brain tissue in non-pathological conditions and in different anatomical sites including the cerebral cortex, thalamus, basal ganglia and hippocampus [28]. In 1938, perineuronal satellitosis was first found on GBM tissue and considered as a form of “secondary structure” and also termed as “perineural growth” [29]. This histological finding, although not specific, has been frequently reported in diffuse gliomas and considered to be the result of the cross-talk existing between neurons and tumor cells [28]. However, since, even in the context of satellitosis associated with diffuse gliomas, it has not yet been clarified whether the glial cells are actually neoplastic in nature or not, we did not consider perineuronal satellitosis as a histological sign of tumor infiltration.

Since the 5th edition of the WHO classification of CNS tumors established some molecular criteria for GBM, IDHwt diagnosis [3,30], one limitation of our study is that molecular analyses were performed for diagnostic purposes only on those cases from our cohort that showed a morphology consistent with a high-grade astrocytic tumor, IDHwt, lacking necrosis and MVP, and in no FLAIR samples. The analysis of the molecular features of GBM IDHwt both in EC and the FLAIR hyperintensity region could be an additional interesting future perspective of this project, as we think that it may have an impact on the histological composition of the latter.

Finally, we believe that a deeper knowledge of the histological composition of the peritumoral area is essential to justify the need for a more or less aggressive surgical resection; accordingly, we encourage further studies with larger cohorts to reach a final agreement on this highly debated topic.

## Figures and Tables

**Table 1:** Differential distribution of the histological features between EC and FLAIR regions.

	EC	FLAIR
**Necrosis and/or MVP**	29/33 (88%)	3/33 (9%)
**High-grade astrocytic tumor lacking necrosis and MVP**	4/33 (12%)	0/33 (0%)
**White matter infiltration**	0/33 (0%)	25/33 (76%)
**Reactive astrogliosis and perineuronal satellitosis**	0/33 (0%)	5/33 (15%)

## Data Availability

All data presented in this article are available from the corresponding author upon reasonable request.
